# How to deal with missing longitudinal data in cost of illness analysis in Alzheimer’s disease—suggestions from the GERAS observational study

**DOI:** 10.1186/s12874-016-0188-1

**Published:** 2016-07-18

**Authors:** Mark Belger, Josep Maria Haro, Catherine Reed, Michael Happich, Kristin Kahle-Wrobleski, Josep Maria Argimon, Giuseppe Bruno, Richard Dodel, Roy W Jones, Bruno Vellas, Anders Wimo

**Affiliations:** Lilly Research Centre, Eli Lilly and Company Limited, Erl Wood Manor, Sunninghill Road, Windlesham Surrey, GU20 6PH UK; Parc Santari Sant Joan de Deu, CIBERSAM, Universitat de Barcelona, Sant Boi de Llobregat, Barcelona, Spain; Eli Lilly and Company, Indianapolis, IN USA; Agencia Qualitat I Avaluacio Sanitaries, Barcelona, Spain; Clinica della Memoria, Department of Neurology and Psychiatry, University of Rome “Sapienza”, Rome, Italy; Department of Neurology, Philipps-University, Marburg, Germany; RICE (The Research Institute for the Care of Older People), Royal United Hospital, Bath, UK; Gerontopole, Toulouse University Hospital, INSERM 1027, Toulouse, France; Division of Neurogeriatrics, Department of Neurobiology, Care Science and Society, Karolinska Institute, Stockholm, Sweden

**Keywords:** Alzheimer’s disease, Cost of illness, Missing data analysis, Missing data mechanisms, Multiple imputation

## Abstract

**Background:**

Missing data are a common problem in prospective studies with a long follow-up, and the volume, pattern and reasons for missing data may be relevant when estimating the cost of illness. We aimed to evaluate the effects of different methods for dealing with missing longitudinal cost data and for costing caregiver time on total societal costs in Alzheimer’s disease (AD).

**Methods:**

GERAS is an 18-month observational study of costs associated with AD. Total societal costs included patient health and social care costs, and caregiver health and informal care costs. Missing data were classified as missing completely at random (MCAR), missing at random (MAR) or missing not at random (MNAR). Simulation datasets were generated from baseline data with 10–40 % missing total cost data for each missing data mechanism. Datasets were also simulated to reflect the missing cost data pattern at 18 months using MAR and MNAR assumptions. Naïve and multiple imputation (MI) methods were applied to each dataset and results compared with complete GERAS 18-month cost data. Opportunity and replacement cost approaches were used for caregiver time, which was costed with and without supervision included and with time for working caregivers only being costed.

**Results:**

Total costs were available for 99.4 % of 1497 patients at baseline. For MCAR datasets, naïve methods performed as well as MI methods. For MAR, MI methods performed better than naïve methods. All imputation approaches were poor for MNAR data. For all approaches, percentage bias increased with missing data volume. For datasets reflecting 18-month patterns, a combination of imputation methods provided more accurate cost estimates (e.g. bias: −1 % vs −6 % for single MI method), although different approaches to costing caregiver time had a greater impact on estimated costs (29–43 % increase over base case estimate).

**Conclusions:**

Methods used to impute missing cost data in AD will impact on accuracy of cost estimates although varying approaches to costing informal caregiver time has the greatest impact on total costs. Tailoring imputation methods to the reason for missing data will further our understanding of the best analytical approach for studies involving cost outcomes.

**Electronic supplementary material:**

The online version of this article (doi:10.1186/s12874-016-0188-1) contains supplementary material, which is available to authorized users.

## Background

Many studies of people with Alzheimer’s disease (AD) determine disease-related costs through cross-sectional analysis or from retrospective databases. Such studies do not account for missing cost data, which can lead to bias and reduce the statistical power to detect effects [1, 2]. Also, they often assume that costs are missing completely at random (MCAR) or missing at random (MAR) rather than missing not at random (MNAR); see Table 1 for a description of these missing data patterns [3]. Although data can be missing for many reasons, dropout of patients with AD dementia from longitudinal studies is a known issue and may not occur completely at random. About 20 % of patients drop out of a longitudinal AD study each year [2] and, in the GERAS observational study [4], 32.4 and 50.6 % of patients discontinued over 18 and 36 months, respectively (data on file). Such dropout can be associated with various patient factors, including poor cognitive performance or impaired functional ability due to worsening disease status, comorbid medical illness, as well as hospitalisation, institutionalisation or death [2, 5]. Loss of the caregiver from the study due to death, illness, increased burden or a change in caregiver will also result in cost and resource use data not being collected.

**Table 1:** Patterns of missing data

Missing data pattern	Description
Missing completely at random (MCAR)	• Data are missing for reasons not related to observed or unobserved variables • Simple statistical approaches to deal with missing data can provide unbiased results
Missing at random (MAR)	• Probability of missingness is related to observed data but not to unobserved data • MAR is the assumption for most imputation methods
Missing not at random (MNAR)	• Missingness is related to unobserved data

It is not possible to distinguish between MAR and MNAR based on observed data

The assumptions made when analysing cost data could have a considerable influence on the conclusions being made. It is, therefore, important to understand the limitations of using a single imputation method for imputing missing cost data (as explored by Oostenbrink & Maiwenn [6]) compared with using different imputation methods depending on the nature of the missingness. It is also important to understand how these decisions compare with other assumptions that are needed when analysing cost data, including the assumptions required for calculating the costs of informal caregiver time. Informal care costs are the largest component of total societal costs associated with the care of community-dwelling patients with AD [4, 7–10]. Various methods are used to collect, categorise and assign a monetary value to informal caregiver time [11–13], and analytical methods such as capping caregiver hours to allow for sleep, including time spent on supervision and using different unit costs based on the working status of the caregiver, can all affect estimates of informal care costs [4, 10].

There is no consensus on the best method to use for dealing with missing cost data and publications on cost of illness studies usually do not provide information that is relevant to understanding the possible impact of the level of missing data and the methods used to deal with it. For example, the analysis of missing data varied across seven published longitudinal studies on the costs of illness associated with AD or dementia that included informal care time and a data collection period of at least 6 months [7, 9, 14–18]. Most studies did not report the amount of missing data, even though the choice of method used to deal with such data may become more important as the level of missing data increases. Several of the studies did not describe how missing data were managed in the cost analysis or whether any sensitivity analyses were performed to examine the potential impact of missing data assumptions on the results. Some studies analysed only patients with complete cost data [7], whereas others used some form of imputation method based on assumptions around missingness, usually that cost data are MAR [9, 14, 15, 17]. However, patients who discontinue from studies due to institutionalisation or death could be considered to have informative dropout and, therefore, data could be defined as MNAR.

The first aim of the present analysis was to explore the effectiveness of different imputation methods for dealing with missing cost data from a prospective observational study (the GERAS study [4]). Our second aim was to compare the use of individual and combined methods of imputing missing total societal cost data. Our third aim was to compare the impact of imputation methods for dealing with missing cost data with the impact of different assumptions on the calculation of caregiver time.

## Methods

### GERAS study

GERAS is an 18-month prospective observational study of costs associated with the care of community-dwelling patients with AD dementia and their caregivers in three European countries (France, Germany, UK) [4]. Full details of the study design, patient characteristics and baseline costs have been reported elsewhere [4]. Briefly, the study enrolled community-dwelling patients aged at least 55 years, meeting the National Institute of Neurological and Communicative Disorders and Stroke/Alzheimer’s Disease and Related Disorders Association criteria for probable AD [19]), with a Mini-Mental State Examination (MMSE) [20] score of ≤26, and presenting within the normal course of care. Caregivers were informal carers who took responsibility for the day-to-day decisions and provision of home care for the patients. Written informed consent was obtained from all participants.

Health care resource use by patients and caregivers was collected using the Resource Utilisation in Dementia (RUD) instrument [21]. Caregiver informal care time was recorded as time spent on basic activities of daily living (ADL, e.g. bathing, feeding), instrumental ADL (e.g. shopping, cooking) and supervision.

Cost data were calculated by country from the resource use information collected for the month before the baseline visit. Monthly costs in Euros (2010 values) were estimated by applying country-specific unit costs for services and products used, with UK costs in pounds sterling being converted to Euros as reported previously [4]. Total societal costs were calculated by combining patient health care costs (including hospitalisations, outpatient visits and medication), patient social care costs (including home care and day centre sessions), caregiver health care costs, and caregiver informal care costs (from time spent giving informal care), each of which were calculated as reported previously [4]. Different methods may be used for estimating informal care time [11], and no one method has been established as the preferred method. For this study, informal care costs were calculated using the higher cost of either caregiver time spent on the patient (excluding supervision time) or caregiver missed work, with the same unit cost being applied to both items. Different unit costs were applied for working and non-working caregivers [4].

### Design

The present analysis evaluated the performance of different imputation methods for dealing with missing cost data for patients with AD dementia through simulations using actual cost data taken from the GERAS study. As 99.4 % of patients reported complete cost data at the baseline assessment, there was a very low level of missing data. The simulations were constructed to look at different patterns and volumes of missing total societal cost data. We have not explored imputation at the cost item level. The simulated datasets were generated using baseline GERAS data where the ‘true’ (actual) cost estimates were known, thus allowing the performance of each imputation method in terms of mean costs and standard errors (SEs) to be compared with known GERAS cost data. As the purpose of this analysis was to test the effectiveness of different methods for dealing with missing cost data, there is no interpretation of the cost data and we do not make any statements about the relationship between missing data and the factors used to generate the simulated datasets.

### Imputation methods

Oostenbrink & Maiwenn [6] compared a number of different naïve and multiple imputation (MI) methods. We explored a similar range of imputation methods to see if Oostenbrink & Maiwenn’s conclusions were supported when using these methods on actual cost data rather than simulated cost data. We present the results for one naïve method (complete case analysis) and one MI method (MI Monte Carlo Markov Chain [MCMC]); the other methods in general gave similar findings.

Among the so-called naïve methods for handling missing data [22], complete case analysis excludes the data from all patients with missing data (i.e. no imputation) and assumes that patients with complete data are representative of those with missing data. However, as patients who do not complete their follow-up are often those with more severe disease, complete case analysis can be expected to underestimate costs. It can also exclude a large proportion of the sample from the analysis.

Imputation methods replace a missing value that is not observed with an estimated value [22]. Multiple imputation is an analytical approach that replaces each missing value with a set of multiple (M > 1) simulated values [6, 23, 24]. The creation of m sets of imputations reflects uncertainty about the true values of the missing data. After the MIs are created, m plausible versions of the complete data exist. In this study, the results of the m analysis are then combined to give one estimate of mean costs.

MCMC algorithms are stochastic and converge to a probability distribution [25]. In this case, the MCMC method converges to the posterior predictive function from which values are drawn to impute the data set. The method assumes arbitrary missing data. In the present analysis, a Jeffrey’s non-informative prior is used, with multiple chains, a 2000 iteration burn-in, and 500 iterations between imputations in a chain. Initial estimates from the MCMC are from an expectation maximisation algorithm. The MI MCMC method assumes that the data are normally distributed.

### Simulation datasets

A brief description of the theoretical generation of missing cost data for the simulations is given below; full details of how the simulation datasets were generated are provided in Additional file 1.

The simulation datasets were derived from the baseline GERAS cost data; 1488 (99.4 %) of the 1497 patients analysed had sufficient resource use information to calculate costs [4]. Total societal costs were used for the simulations and patient baseline characteristics from the GERAS study were used in the creation of the simulation datasets.

Algorithms were created to generate 12 different simulation datasets with three different mechanisms of missing cost data (MCAR, MAR and MNAR), each at different volumes of missing total cost data (10, 20, 30 and 40 %), the potential range of missing data from longitudinal studies of people with AD. In addition, two further simulation datasets were generated (GERAS-1, GERAS-2), where the pattern and volume of missing data reflected the observed patient dropout rate during 18 months of follow-up in the GERAS study.

MCAR datasets were created using a random number generator to select patients to be missing and using values that gave the required volume of missing data (10–40 %).

MAR scenarios assume that some known factors predict which data are MAR. Our simulations were based on a theoretical example of missing cost data being associated with two baseline characteristics: patient functional and cognitive status at baseline (i.e. Alzheimer’s Disease Cooperative Study of Activities of Daily Living Inventory, ADCS-ADL [26] and MMSE scores). Thus, MAR datasets were generated by assigning missing cost data based on a specific score for these two combined variables, with the score being set to achieve the required volume of missing data.

MNAR scenarios assume that some unknown factor(s) cause the missing data. However, a factor must be used to generate the theoretical MNAR dataset for the simulations; we used baseline GERAS total cost data above a specific value (which was set based on the volume of missing data required for each dataset) to assign patients as having missing costs.

In the real world, missing data comprise a mixture of different patterns of missingness, with some patient data being MCAR, and other data being MAR or MNAR. Because this was likely to be the case with the GERAS cost data, we generated two simulation datasets, GERAS-1 and GERAS-2, to more closely represent the structure of real missing data. First, we used the rate of patient discontinuation at the GERAS 18-month assessment and the reasons for study discontinuation to give both a level of missing cost data and different patterns of missing cost data for specific groups of patients. Three different reasons for missing data were defined: (1) missing due to patient institutionalisation; (2) missing due to patient death; and (3) missing because the patient had left the study (i.e. lost to follow-up). The 18-month data from GERAS showed that 15 % of patients were institutionalised, 6 % had died, and 12 % were lost to follow-up, which gave 33 % of missing cost data. Two simulation datasets were then generated based on this missing data pattern. In dataset GERAS-1, patients assigned as being institutionalised were generated as MAR based on observed data (e.g. MMSE and ADCS-ADL scores and caregiver time), while in dataset GERAS-2, institutionalised patients were assumed to be MNAR due to unobserved data; these data were generated using total caregiver time at baseline as the unknown factor (which was not used as part of the imputation methods). In both GERAS-1 and GERAS-2, patients assigned as being lost to follow-up were assumed to be MCAR and were generated using a random number generator, while death was generated as MAR using a combination of observed factors (baseline MMSE and ADCS-ADL scores, and patient age).

### Analyses

All analyses were carried out using SAS 9.2 (SAS Institute, Cary, North Carolina, USA).

The simulation datasets generated for the 12 mechanisms of missing data at different volumes of missing data and the two patterns of real missing data (GERAS-1 and GERAS-2) were each run against the two different imputation methods described previously (complete cases, MI MCMC). In addition, the impact of applying a combination of imputation methods to missing data was explored using two different combination imputation scenarios for the GERAS-1 and GERAS-2 datasets. In Combination Scenario A, missing cost data for patients lost to follow-up (assumed to be MCAR) were imputed using the naive method of grouped means imputation, which uses the mean costs from non-missing observations for each MMSE severity group at baseline (mild, moderate; and moderately severe/severe AD dementia based on MMSE scores of 21–26, 15–20 and <15, respectively) to impute missing data within each severity group; missing cost data for patients institutionalised (assumed to be MAR) were imputed using the MI MCMC method (including the factors MMSE score, ADCS-ADL score and caregiver time); and missing cost data for patients who died (assumed to be MAR) were imputed using the MI MCMC method (including the factors patient age, MMSE score and ADCS-ADL score). In Combination Scenario B, a fixed cost (€2940 per month, cost of institutionalisation) was used for missing cost data for institutionalised patients (assumed to be MNAR); the imputation methods used for missing cost data for patients who died or were lost to follow-up were the same as for Combination Scenario A.

To avoid the results being influenced by one particular set of data, we performed each imputation scenario 1000 times on slightly different sets of data generated by random selection with replacement for each of the 14 simulation datasets generated. The estimates of these costs from the 1000 iterations were combined and compared with the ‘true’ cost estimates from the complete sample with no missing cost data.

Six outcome measures were used to assess the performance of the imputation methods:

1. *mean costs* (in Euros) for both the ‘true’ (actual) mean cost from the complete sample and the estimated mean cost from the imputed simulation datasets;
2. *absolute and relative bias*: calculated as the difference in mean costs between the actual costs for the GERAS complete sample dataset and the estimated cost for the analysed sample; i.e. absolute bias was calculated as (estimated cost−actual cost), while relative bias (%) was calculated as [(estimated−actual cost)/actual cost × 100]; these values should be as close to zero as possible (negative values indicate underestimation of costs, whereas positive values reflect overestimation of costs);
3. *sampling standard error (SSE)* for the estimator, being the standard deviation (SD) of the mean costs of the 1000 iterations;
4. *sampling average of the standard error estimator (SEE)*, being the mean of the SEs of the 1000 iterations;
5. *ratio of SEE/SSE*, which can be considered a measure of whether the analysis provides an adequate estimate of the SE of the dataset, and reflects the extent to which the SEE approaches the SSE (calculated as the ratio SEE/SSE);
6. *sampling coverage probability* (CP) of the 95 % confidence interval (CI), being the proportion of iterations for which the 95 % CI includes the ‘true’ mean costs. If the CP is high, it means that the estimated CI will generally include the ‘true’ mean of the costs; a low CP indicates it is less likely to contain the ‘true’ mean. Low CP scores are an indication that the estimated mean is a poor estimate of the ‘true’ mean.

For each analysis, the SSE can be considered as the ‘true’ SE and should be larger than the SE of the complete sample to indicate the additional uncertainty due to imputation of missing data.

#### Sensitivity analysis

Two sensitivity analyses were conducted to investigate the impact of (1) using imputation methods that were dependent on baseline characteristics, when unmeasured confounders were present, and (2) the assumption of normality in smaller sample sizes. Further details of these sensitivity analysis methods can be found in Additional file 2.

### Alternative methods for costing caregiver time

To put the impact of using different imputation methods for dealing with missing cost data into context, we assessed the effects of using different methods to calculate informal care costs on the estimate of total societal costs. In the base case analysis, which was the same as the ‘true’ mean cost used for the simulations, total societal costs were calculated excluding caregiver supervision hours in the opportunity cost approach. Alternative total societal cost estimates were generated where: (1) caregiver supervision time was included, (2) replacement costs were used instead of opportunity costs, and (3) only caregiver time for working caregivers was included for caregiver informal costs (i.e. caregiver time for non-working caregivers was not costed; see Wimo et al. [4] for details).

## Results

### GERAS baseline data

Of the 1497 patients in the GERAS study analysed at baseline, mean (SD) age was 77.6 (7.66) years, 54.8 % were female, and the mean (95 % CI) MMSE score was 17.4 (17.1; 17.7) as reported previously [4].

Total societal costs were available for 1488 (99.4 %) patients, with a mean monthly cost of €2101 (95 % CI: €1980–2222).

### Simulations

**Fig. 1:**
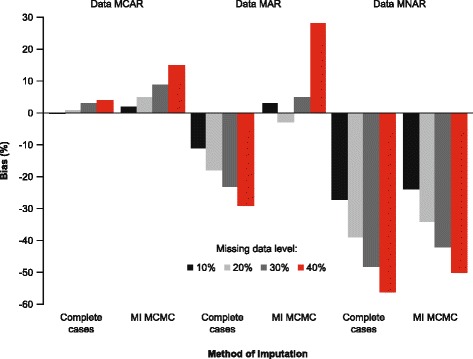
Bias in mean costs for imputation methods for different mechanisms and levels of missing data. Abbreviations: MCAR: missing completely at random; MAR: missing at random; MNAR: missing not at random; MCMC: Markov Chain Monte Carlo; MI: multiple imputation

Figure 1 shows the % bias in mean costs for the two imputation methods (complete cases, MI MCMC) under the different mechanisms and different volumes of missing data. In general, the % bias increased as the volume of missing data increased from 10 to 40 %. When data were MCAR, the naïve imputation (complete cases) performed as well as the more complicated MI MCMC method; for complete cases, the rate of bias ranged from −0.03 % with 10 % missing data to 4 % with 40 % missing data; for MI MCMC, the rate of bias ranged from 2 % with 10 % missing data to 15 % with 40 % missing data.

When data were MAR, the MI MCMC method performed better at 10–30 % missing data (bias ranged from −3 to 5 %) when compared with the complete case analysis (bias ranged from −11 to –23 %). However, when the level of missing data reached 40 %, the percent bias in mean costs increased for the MI MCMC method (28 %), and was of a similar magnitude to the complete cases method (−29 %) although in the opposite direction.

When data were MNAR, all imputation methods performed poorly and produced high levels of percent bias in mean costs, showing an underestimation of costs: complete cases bias ranged from −27 % at 10 % missing data to −56 % at 40 % missing data; MI MCMC bias ranged from −24 % at 10 % missing data to −50 % at 40 % missing data.

Table 2 summarises the results for the simulations for the three different mechanisms of missing data for complete cases and MI MCMC. For the MCAR and MAR simulations, the SSE with the MI MCMC method was greater than the SEE of the complete sample (reflecting greater variability) at all levels of missing data; this was not seen for the complete cases method. For the MNAR simulations, the SSE was lower than the SE of the ‘true’ mean, whereas you would expect it to be higher based on the uncertainty introduced with the additional missing data. The results of the simulations for the different mechanisms of missing data for all naïve and MI methods examined are available on request.

**Table 2:** Summary statistics of simulations^a^ with missing data patterns (MCAR, MAR, MNAR)

Imputation method for missing data pattern	10 % missing					20 % missing					30 % Missing					40 % missing				
	Mean cost (€)	Bias (%)	SSE	SEE	CP	Mean cost	Bias (%)	SSE	SEE	CP	Mean cost	Bias (%)	SSE	SEE	CP	Mean cost	Bias (%)	SSE	SEE	CP
Complete sample	2102	–	59	61	–	–	–	59	61	–	–	–	59	61	–	–	–	59	61	–
*MCAR*																				
Complete cases	2102	−0.7 (−0.03 %)	63	64	1.00	2121	18 (0.9 %)	68	69	1.00	2156	53 (3 %)	75	76	1.00	2181	79 (4 %)	87	87	0.99
MI MCMC	2150	48 (2 %)	68	58	1.00	2218	115 (5 %)	78	57	0.46	2300	198 (9 %)	93	57	0.03	2410	308 (15 %)	118	57	0.00
*MAR*																				
Complete cases	1871	−231 (−11 %)	56	57	0.00	1723	−379 (−18 %)	54	55	0.00	1624	−478 (−23 %)	59	59	0.00	1499	−603 (−29 %)	59	61	0.00
MI MCMC	2158	55 (3 %)	112	57	0.76	2039	−64 (−3 %)	83	48	0.65	2218	116 (5 %)	202	52	0.38	2683	581 (28 %)	417	69	0.09
*MNAR*																				
Complete cases	1544	−558 (−27 %)	27	28	0.00	1291	−812 (−39 %)	22	22	0.00	1096	−1007 (−48 %)	20	19	0.00	929	−1173 (−56 %)	17	16	0.00
MI MCMC	1602	−501 (−24 %)	28	26	0.00	1383	−720 (−34 %)	22	19	0.00	1212	−890 (−42 %)	20	15	0.00	1043	−1059 (−50 %)	19	11	0.00

% bias was calculated as (estimated−actual)/actual cost × 100), where actual cost was the mean cost for the complete sample

*Abbreviations*: *CP* coverage probability, *MCMC* Markov Chain Monte Carlo, *MI* multiple imputation, *SEE* standard error estimate, *SSE* sampling standard error

^a^1000 simulations and sample size 1497 for different levels of missing data (10–40 %)

Table 3 shows how the different imputation methods, including both combination imputation scenarios, performed when the cost data were generated based on the actual level of missing cost data at the 18-month assessment of the GERAS study [33 % missing data in total, due to institutionalisation (15 %), death (6 %) and lost to follow-up (12 %)]. For dataset GERAS-1, the MI MCMC method performed as well as Combination Scenario A, bias being −3 % for both methods. In comparison, the complete cases method showed a higher level of bias (−7 %). Although both the MI MCMC and Combination Scenario A imputation methods gave good estimates of the mean costs (i.e. −3 % bias) for the GERAS-1 dataset, they underestimated the SE because the SEE/SSE ratios were 0.64 and 0.66, respectively.

**Table 3:** Summary statistics of simulations^a^ with missing data pattern reflecting GERAS study data at 18 months^b^

	GERAS-1^c^						GERAS-2^d^					
Imputation method	Mean cost (€)	Bias (%)^e^	SSE	SEE	SEE/SSE	CP	Mean cost (€)	Bias (%)^e^	SSE	SEE	SEE/SSE	CP
Complete sample	2101	–	64	62	0.97	–	2103	–	60	61	1.02	–
Naïve imputation method												
Complete cases	1957	−144 (−7 %)	67	66	0.99	0.38	1689	−414 (−20 %)	52	54	1.04	0.00
Multiple imputation method												
MI MCMC	**2037**	**−64 (−3 %)**	**74**	**47**	**0.64**	**0.70**	1969	−134 (−6 %)	77	41	0.53	0.22
Combination of imputation methods												
Combination Scenario A^f^	**2044**	**−57 (−3 %)**	**71**	**47**	**0.66**	**0.73**	1947	−157 (−7 %)	69	41	0.59	0.12
Combination Scenario B^g^	2296	195 (9 %)	62	49	0.79	0.02	**2075**	**−28 (−1 %)**	**49**	**42**	**0.86**	**0.87**

Numbers in bold text show the imputation method(s) that perform the best (lowest bias) for each of the two datasets (GERAS-1 and GERAS-2)

*Abbreviations*: *CP* coverage probability, *MAR* missing at random, *MCAR* missing completely at random, MCMC Markov Chain Monte Carlo, *MI* multiple imputation, *MNAR* missing not at random, *SEE* standard error estimate, *SSE* sampling standard error

^a^1000 simulations and sample size 1497

^b^Data missing for 33 % patients at 18 months: 15 % patients institutionalised, 6 % died, 12 % lost to follow-up

^c^GERAS-1: assumed patients institutionalised were based on a predictive equation (i.e. data MAR)

^d^GERAS-2: assumed patients institutionalised if their caregiver time was >470 h/month (i.e. data MNAR)

^e^% bias was calculated as ((estimated−actual)/actual cost × 100), where actual cost was the mean cost for the complete sample

^f^Combination Scenario A: patients lost to follow-up (data MCAR) had costs imputed using group means method, patients institutionalised (data MAR) were imputed using MI MCMC method (including factors MMSE, ADCS-ADL and caregiver time), and patients who died (data MAR) had costs imputed using the MI MCMC method (including factors MMSE, patient age and ADCS-ADL)

^g^Combination Scenario B: same imputation methods as Combination Scenario A, but a fixed cost (€2940 per month) was used for patients who were institutionalised (data MNAR)

For dataset GERAS-2, the MI MCMC method performed better than the complete cases method (Table 3). A better approach was to use a combination of imputation methods, especially Combination Scenario B, which had a low level of bias (−1 %), even though it still underestimated the SE (i.e. the SSE was 49, which was lower than 61, the SE for the complete sample). Combination Scenario B gave a lower bias (−1 %) than the MI MCMC method (−6 %), and the SEE/SSE ratio was superior (0.86 for Combination Scenario B versus 0.53 for MI MCMC).

#### Sensitivity analyses

Findings from the sensitivity analyses using other naïve methods and MI methods were consistent with the main conclusions presented in this paper (see Additional file 2).

### Cost estimates using alternative methods for costing caregiver time

To put the assumptions around the different imputation methods into context, cost estimates were calculated using different assumptions on how to include caregiver time into the estimate of total societal costs. The base case scenario (where separate unit costs were used for working and non-working caregivers and supervision time was excluded in the opportunity cost approach), mean monthly total societal costs for the whole cohort were €2101 (95 % CI: €1980–2222). When supervision time was included, mean monthly total societal costs were €2995 (95 % CI: €2832–3158), representing a 43 % increase from the base case estimate. When a single unit cost was used for caregiver time (i.e. same unit cost for working and non-working caregivers; supervision time included), mean monthly total societal costs were €2707 (95 % CI: €2575–2839), corresponding to a 29 % increase from the base case estimate. An alternative approach was to not include costs of informal care when the caregiver is not working for pay; in this analysis, mean monthly total societal costs were €1597 (95 % CI: €1444–1749), giving a−24 % difference from the base case estimate.

## Discussion

Given that the volume of missing data and the methods used to deal with missing data have been poorly reported in previous cost studies in AD, our results showed the importance of being able to understand the reasons for missing data from prospective studies (including why patients discontinue from studies) and how gathering informative data about study discontinuation can lead to better tailoring of methods for applying costs. Furthermore, we put these findings into context and demonstrated that the method of quantification and costing of informal caregiver time has the biggest impact on cost estimates, way above that of the different methods for handling missing data.

Having a nearly complete set of baseline cost data for the large patient sample in the GERAS study allowed us to simulate costs based on real-life data, which have much greater variability and distribution than purely theoretical data. Also, it allowed us to compare the simulated costs with the ‘true’ costs used to simulate the missing data, thereby providing a measure of the performance (bias, accuracy and coverage) of the different imputation methods. Our findings, therefore, extend previous analyses of missing cost data based on simulations of purely theoretical datasets (e.g. Oostenbrink and Maiwenn [6]), which have a low level of bias because they do not include the randomness, variability and inconsistencies of real-world data.

In our simulations, we explored how different imputation methods performed under the different mechanisms of missing data with increasing volumes of missing data. Our findings confirm those reported previously in the theoretical simulation study by Oostenbrink and Maiwenn [6]. We showed that complete case analysis performed as well as MI MCMC for missing cost data assumed to be MCAR. Regardless of the imputation method used, the magnitude of bias increased as the volume of missing data increased. However, in prospective AD studies, it is difficult to assume or prove that data are MCAR unless there are very small amounts of missing data. Thus, previous cost of illness studies in AD that assumed data were MCAR and restricted analyses to study participants with complete data (complete case analysis) [7] could have given biased results, leading to inappropriate conclusions. It is more realistic to assume that data are MAR (with known factors contributing to the missing data). Our analyses showed that MI MCMC performed well for this mechanism of missing data. However, when missing data were assumed to be MNAR (i.e. the trigger for missingness is unknown [3]), all imputation methods performed poorly (i.e. had high levels of bias) and this increased as the missing data level increased to 40 %.

In reality, it is known that missing data occur for many different reasons, especially in studies involving elderly people [27]. One-third of patients with AD dementia dropped out during the 18-month follow-up period of the GERAS study because they died, were institutionalised or were lost to follow-up for other reasons including unavailability of the caregiver. Thus, there were at least three distinct reasons for study discontinuation and, therefore, for not having cost data. In many cost studies, death and institutionalisation are used as end points rather than as missing data, with cost set to zero after death and a fixed cost for institutionalisation. For this analysis, however, we used death and institutionalisation as reasons for having missing data, which enabled the simulation of different assumptions about missing data mechanisms.

By creating datasets that included actual volume and patterns of reasons for discontinuation, we showed that a single imputation method, MI MCMC, was acceptable when the data were MCAR and/or MAR (as in the GERAS-1 dataset). However, when some of the data were MNAR (as in the GERAS-2 dataset) and when using a combination of imputation methods, Scenario B performed better than the individual approaches because it mirrored more closely how the missing data were generated. Combination Scenario A performed better on the GERAS-1 dataset but was similar to the MI methods.

Our analyses showed that it is important to understand the reasons why patients discontinue from a study and the reasons for missing data because it can inform us about the most appropriate imputation methods to use to reduce the biases due to missing data. Using a combined approach for missing data reasons (MCAR, MAR and MNAR) provides the opportunity to minimise the impact of data that are MNAR and the ineffectiveness of imputation methods to deal with this. For example, if 30 % of data are missing overall but only 10 % of these are MNAR, then using an imputation method that assumes the data are MAR will produce less bias than when the proportion of MNAR is 20 %. Kaambwa et al. [28] came to a similar conclusion in their exploration of how different methods for analysing missing data from an observational study were influenced by using extra information obtained during data collection about why data were missing. Thus, reasons for missingness should be collected during prospective studies in order to be informative in cost analyses. This is particularly important for clinical trials, when reasons for missing data may differ depending on the type of treatments administered. This would be applicable not just for AD, but also for other chronic diseases, especially those where a caregiver is also required.

We ran further simulations to consider the impact of unmeasured confounders (see Additional file 2). For imputation methods that relied on knowing which factors were associated with both missing data and costs, we showed that performance was affected if this factor was excluded in the imputation method (as discussed by Spratt et al. [29]). We found that use of the correct combined method could reduce this effect.

In our analyses for imputing missing data, we assumed that the cost data were normally distributed. This is generally not a problem for large datasets like GERAS, where you can assume the central limit theorem, which states that as a dataset gets larger you can assume normality even if the data have a skewed distribution [30]. However, bias due to missing data generally increases with smaller datasets. The MI method used in our analysis assumes normality. However, if working on smaller datasets, MI methods that do not assume that the data are normally distributed (e.g. MI predictive mean matching regression [PMMR] and MI propensity score) should be considered. As part of our sensitivity analysis (see Additional file 2), MI PMMR performed better than the MI regression method as the volume of missing data increased, for both relative bias and estimating the SE. The assumption of normality for the imputation of missing data, however, does not negate the requirement to understand the distributional properties of the cost data (with imputed values) when conducting further statistical analysis.

To gain a better understanding of the relative impact on total societal cost estimates of using different imputation methods for dealing with missing cost data, we estimated total societal costs using different methods for calculating informal care costs. The results showed that decisions made about calculating the costs of caregiver informal care time have a much bigger impact on total societal cost estimates than decisions made about the methods used for handling missing data, at least with the volumes and patterns of missing data used in the present analyses. Various methodological issues that need to be considered when calculating informal care costs include the instrument used to collect data on informal care time and how informal care hours are categorised (i.e. reliability of data capture and use of standardised methods, such as the RUD), what informal care hours are costed (i.e. whether or not supervision time is included), the method used for applying costs (e.g. opportunity versus replacement cost methods) [6], and the valuation of unit costs (e.g. source of unit costs, whether they are country specific). In the present study, the different imputation methods for dealing with missing data had minimal impact on total societal cost estimates compared with including supervision time (which increased total costs by 46 %) or applying different costs for caregiver time (which changed total costs by 24–29 %). Thus, transparency in approaches to costing informal care will enable more accurate cost comparisons, which will be particularly important when assessing treatment effects.

Our analyses have several potential limitations. First, the simulations are dependent on the method used and the assumptions made. Simulation studies are complex to perform and each of the many decisions that have to be made at each stage of the process requires careful consideration [31]. Simulation datasets can be generated in many different ways using different factors. However, if a different set of factors is used to create the dataset, this may impact on the outcomes. For example, cost was used to generate the MNAR dataset. Also, some of the imputation methods used MMSE and ADCS-ADL scores as factors in the model, and these factors are associated with costs, such that costs increase as cognitive and functional ability scores worsen [4, 8, 9, 14–16, 18, 32].

We applied a simple mechanism to generate missing cost data based on only a small number of patient characteristics. In reality, however, missing cost data are likely to be influenced by multiple patient characteristics. We have not explored if these methods are affected by the number of patient factors that determine the missingness of the data.

For the purposes of this analysis, we looked at methods used to estimate costs at a specific point in time (i.e. after 18 months of follow-up). That is, we did not apply methods which take account of missing cost data at each visit. It may be important to model costs throughout the follow-up period through repeated measure models. In such circumstances, other imputation methods, such as last observation carried forward or mixed-effect model repeated measures, would need to be considered alongside the MI methods used. However, our conclusions that bias can be reduced when the different mechanisms of missingness can be identified are somewhat overshadowed by the effect of the decisions made about calculating the costs of caregiver time. These have a much more significant impact on societal costs than the choice of imputation methods used.

Future work using data generated from the GERAS study could include analyses to understand the differences by country, to assess whether country-specific methods for missing data need to be applied. It would be interesting to examine whether the approach of using combinations of imputation methods based on the different reasons for why missing data occur can be applied to other disease area costs. In addition, further research is needed on how to apply these methods for handling missing data when individual measures or items of measures are missing.

## Conclusions

Missing data is a complex issue when estimating the cost of illness, as exemplified in this analysis from the GERAS study of patients with AD dementia. First, the bias caused by missing data can be reduced by a better understanding of the causes of the missing data. Where there are different reasons for the missing data, consideration should be given to the use of different imputation methods. The use of single imputation techniques can be improved by using the missingness information to tailor imputation rules, resulting in more accurate cost estimates. Second, in the context of other assumptions made for estimating costs of AD (with missing data similar to the GERAS profile), then methods for costing informal caregiver time have a much larger impact on the cost estimates compared with assumptions made on which imputation method to use. The current analysis from the GERAS study provides important recommendations for the planning of studies involving cost outcomes, both observational and interventional, when there may be different reasons for dropout between cohorts. The same analytical approach could be useful for studies of other chronic diseases that require long-term follow-up and have a high dropout rate. Limiting the amount of uninformative missing data will allow a simpler approach to imputation, as fewer assumptions will be required.

## Abbreviations

AD, Alzheimer’s disease; ADCS-ADL, Alzheimer’s disease cooperative study of activities of daily living; CI, confidence interval; CP, coverage probability; MAR, Missing at random; MCAR, Missing completely at random; MCMC, Markov chain Monte Carlo; MI, Multiple imputation; MMSE, Mini-mental state examination; MNAR, Missing not at random; RUD, Resource use in dementia; SD, Standard deviation; SE, Standard error; SEE, Sampling average of the standard error estimator; SSE, Sampling standard error

## Additional files

Additional file 1: Details of the rationale used to generate datasets to be used in the simulation. (DOCX 19 kb) Additional file 2: Details of the sensitivity analyses performed and results obtained. (DOCX 770 kb)
