# Supplementary material for: How to deal with missing longitudinal data in cost of illness analysis in Alzheimer’s disease—suggestions from the GERAS observational study
Source: BMC Med Res Methodol. 2016 Jul 18;16:83. doi: 10.1186/s12874-016-0188-1 (PMC4950752; doi:10.1186/s12874-016-0188-1)
Supplement: Additional file 1: — Details of the rationale used to generate datasets to be used in the simulation. (DOCX 19 kb) [file 12874_2016_188_MOESM1_ESM.docx]

**How to deal with missing longitudinal data in cost of illness analysis in Alzheimer’s disease – suggestions from the GERAS observational study**

*BMC Medical Research Methodology*

**Additional file 1**

**Details of the rationale used to generate datasets to be used in the simulations**

1. *Generation of datasets for missing completely at random (MCAR)*

A random number from a normal distribution X~N(0,1) is assigned to each patient in the cost dataset. Patients are then allocated a missing cost variable if the random number exceeds 1.265, which will result in 10% of the patients having a missing total cost assigned to them. To assign 20% of the patients with missing total costs, then X>0.845. If X>0.525 then 30% of patients are assigned a missing total cost, while X>0.24 will assign 40% of patients with missing total costs.

1. *Generation of datasets for missing at random (MAR)*

To assign missing total cost data based on a MAR mechanism, we have assumed that missing data are associated with two of the patient’s baseline characteristics. For the purposes of these simulations, we have chosen to use the Alzheimer’s Disease Cooperative Study of Activities of Daily Living Inventory (ADCS-ADL) score and the Mini-Mental State Examination (MMSE) score to be associated with missing cost data (note: any combination of variables could have been chosen to generate the missing data patterns; the important point is that we know which variables are associated with generating missing data).

To generate data MAR, it is necessary to first establish a relationship between the patient characteristics and missing cost indicator. For our simulations, we have chosen the following relationship:

Missing cost Indicator (MCI) = ((ADCS-ADL score)***3**) + (**0.25***(MMSE score))

Based on the distribution of the MCI, lower limits for 10–40% for the distribution are calculated.

In these simulations, patients are then assigned missing total cost data if:

1. MCI <57.6 (10% of patients are assigned missing total costs)
2. MCI <88.6 (20% of patients are assigned missing total costs)
3. MCI <113.1 (30% of patients are assigned missing total costs)
4. MCI <132.1 (40% of patients are assigned missing total costs)

1. *Generation of datasets for missing not at random (MNAR)*

To assign missing cost data based on a MNAR mechanism, we have chosen for these simulations to base this on costs missing if the actual baseline costs exceeded a specific value. Cut-off values were selected to ensure 10%, 20%, 30% and 40% of the patients were assigned missing total cost data.

Based on the distribution of GERAS baseline total costs (baseline total cost (BTC)) patients were then assigned missing total cost data for the simulation datasets if the following conditions were met:

1. BTC >4181.83 (10% of patients are assigned missing total costs)
2. BTC >3032.01 (20% of patients are assigned missing total costs)
3. BTC >2330.5 (30% of patients are assigned missing total costs)
4. BTC >1888.78 (40% of patients are assigned missing total costs)
5. *Generation of datasets for missing data pattern for GERAS-1 datasets*

Simulation datasets were created to reflect a pattern of missingness that had similar volume to the missing data observed after 18 months of follow-up in GERAS, and was based on different mechanisms of missing data. For these simulations, it was decided that there would be three reasons why a patient would not have data available for total costs. To reflect the information available from the GERAS longitudinal data, we based these on: 1) patients who had been institutionalised; 2) patients who had died; and 3) patients who had decided to withdraw from the study.

A two-step approach was undertaken to create the missing data:

The GERAS 18-month data showed that 15% of patients left the study due to being institutionalised, 6% of patients died during the 18-month follow-up period, while a further 12% withdrew from the study, resulting in 33% of patients having no total cost data. This information was used to create a simulation dataset, where each reason for discontinuing from the study had its own mechanism of missingness.

Patients withdrawing from the study were assumed to be MCAR, whereas patients with missing cost data due to institutionalisation were assumed to be MAR; patients missing due to death were also assumed to be MAR.

The following relationships were derived for missing cost data for:

1. Institutionalisation

Missing(Inst) = (Total caregiver time) + **5***(MMSE score) + **10*** (Total ADCS-ADL score)

Patients are assigned missing total cost data if Missing(Inst) >891. This will assign 15% of the simulation dataset with missing cost data due to institutionalisation.

1. Death

Missing(death) = **1.5***(Patient age) + **0.5***((Patient age)^2^) - **3***(MMSE score) - (Total ADCS-ADL score)

Patients are assigned missing total cost data if Missing(Death) >3886. This will assign 6% of the simulation dataset with missing cost data due to death.

1. Patient withdrawals

Patients are assigned missing total cost data if random number from N(0,1) is greater than 1.175. This will assign 12% of the simulation dataset with missing cost data due to patients withdrawing from the study.

1. *Generation of datasets for missing data pattern for GERAS-2 datasets*

Simulation datasets were created to reflect a pattern of missingness that had similar volume to the missing data observed after 18 months of follow-up in GERAS, and was based on different mechanisms of missing data. For these simulations, it was decided that there would be three reasons why a patient would not have data available for total costs. To reflect the information available from the GERAS longitudinal data, we based these on 1) patients who had been institutionalised; 2) patients who had died; and 3) patients who had decided to withdraw from the study.

A two-step approach was undertaken to create the missing data:

The GERAS 18-month data showed that 15% of patients left the study due to being institutionalised, 6% of patients died during the 18-month follow-up period, while a further 12% withdrew from the study, resulting in 33% of patients having no total cost data. This information was used to create a simulation dataset, where each reason for discontinuing from the study had its own mechanism of missingness.

Patients withdrawing from the study were assumed to be MCAR, while patients with missing cost data due to institutionalisation were assumed to be MNAR; patients missing due to death were also assumed to be MAR.

The following relationships were derived for missing cost data for:

1. Institutionalisation

Patients are assigned missing total cost data if total caregiver time is greater than 470 hours per month. This will assign 15% of the simulation dataset with missing cost data due to institutionalisation. (Note: when applying imputation methods to this simulation dataset the variable total caregiver hours is assumed to be unknown.)

1. Death

Missing(death) = **1.5***(Patient age) + **0.5***((Patient age)^2^) - **3***(MMSE score) - (Total ADCS-ADL score)

Patients are assigned missing total cost data if Missing(Death) >3886. This will assign 6% of the simulation dataset with missing cost data due to death.

1. Patient withdrawals

Patients are assigned missing total cost data if random number from N(0,1) is greater than 1.175. This will assign 12% of the simulation dataset with missing cost data due to patients withdrawing from the study.
