# Supplementary material for: How to deal with missing longitudinal data in cost of illness analysis in Alzheimer’s disease—suggestions from the GERAS observational study
Source: BMC Med Res Methodol. 2016 Jul 18;16:83. doi: 10.1186/s12874-016-0188-1 (PMC4950752; doi:10.1186/s12874-016-0188-1)
Supplement: Additional file 2: — Details of the sensitivity analyses performed and results obtained. (DOCX 770 kb) [file 12874_2016_188_MOESM2_ESM.docx]

**How to deal with missing longitudinal data in cost of illness analysis in Alzheimer’s disease – suggestions from the GERAS observational study**

*BMC Medical Research Methodology*

**Additional file 2**

**Details of the sensitivity analyses performed and results obtained**

### Sensitivity analysis methods

Missing data classified as missing at random (MAR) may be influenced by unmeasured confounders, which would then follow more of a missing not at random (MNAR) process. This may have an impact on the imputation method used, especially methods which use patient characteristics data. In the first sensitivity analysis, alternative imputation scenarios were run to assess the effect of missing data being generated due to unmeasured confounders. To assess the impact of unmeasured confounders, we looked at the imputation methods that were dependent on identifying the factors associated with the missing data; i.e., the predicted regression method, the four multiple imputation (MI) methods, and the two combination imputation scenarios. When applying these methods, it was assumed that the baseline Alzheimer’s Disease Cooperative Study of Activities of Daily Living Inventory (ADCS-ADL) score (used to generate the missing data pattern) was not available for use as part of the imputation method, and so acted as an unmeasured confounder. These simulations were run on the GERAS-1, GERAS-2 and MAR datasets.

For many of the simulations, we have used methods that have assumed that the cost data were normally distributed. The large sample size allowed normality assumptions based on the central limit theorem; however, it is important to understand the impact that these methods have on the imputation of cost data from smaller sample sizes. In the second sensitivity analysis, the effect of sample size was assessed for selected imputation scenarios. The primary simulations were based on a sample size of 1497 patients (i.e., the full GERAS study cohort at baseline). In two additional sets of simulations, the sample size was 500 patients (chosen to reflect the size of the country-specific analysis in the GERAS study), and 100 patients (chosen to reflect the approximate size of the disease severity categories within each country in the GERAS study and the likely sample size for cost of illness studies). These simulations were also run on the GERAS-1 and GERAS-2 datasets.

**Results of sensitivity analyses**

### Unmeasured confounders

Supplemental Figure 1 presents the effect of unmeasured confounders on % bias in mean costs for selected imputation methods with the GERAS-1 and GERAS-2 datasets. This shows that for dataset GERAS-1, unmeasured confounders had a limited impact on the performance (% bias) of the imputation methods used; performance was similar to the methods that did not account for unmeasured confounding (values in brackets at the top of Supplemental Figure 1). However, for dataset GERAS-2, unmeasured confounders increased the % bias for the predicted regression and four MI methods by 2–5% compared with the primary simulations, but the combination methods of imputation were relatively unaffected. The impact of unmeasured confounders on the MAR datasets (Supplemental Figure 2) showed that the % bias increased for the predicted regression, MI propensity score and MI predictive mean matching regression (PMMR) methods, and that these increased with increasing missing data. However, the results for the MI Monte Carlo Markov Chain (MCMC) and MI regression methods were relatively unaffected by unmeasured confounders, and showed a smaller increase in bias as the volume of missing data increased.

**Supplemental Figure 1** Effect of unmeasured confounders on percentage bias in mean costs for selected imputation methods on datasets GERAS-1 and GERAS-2


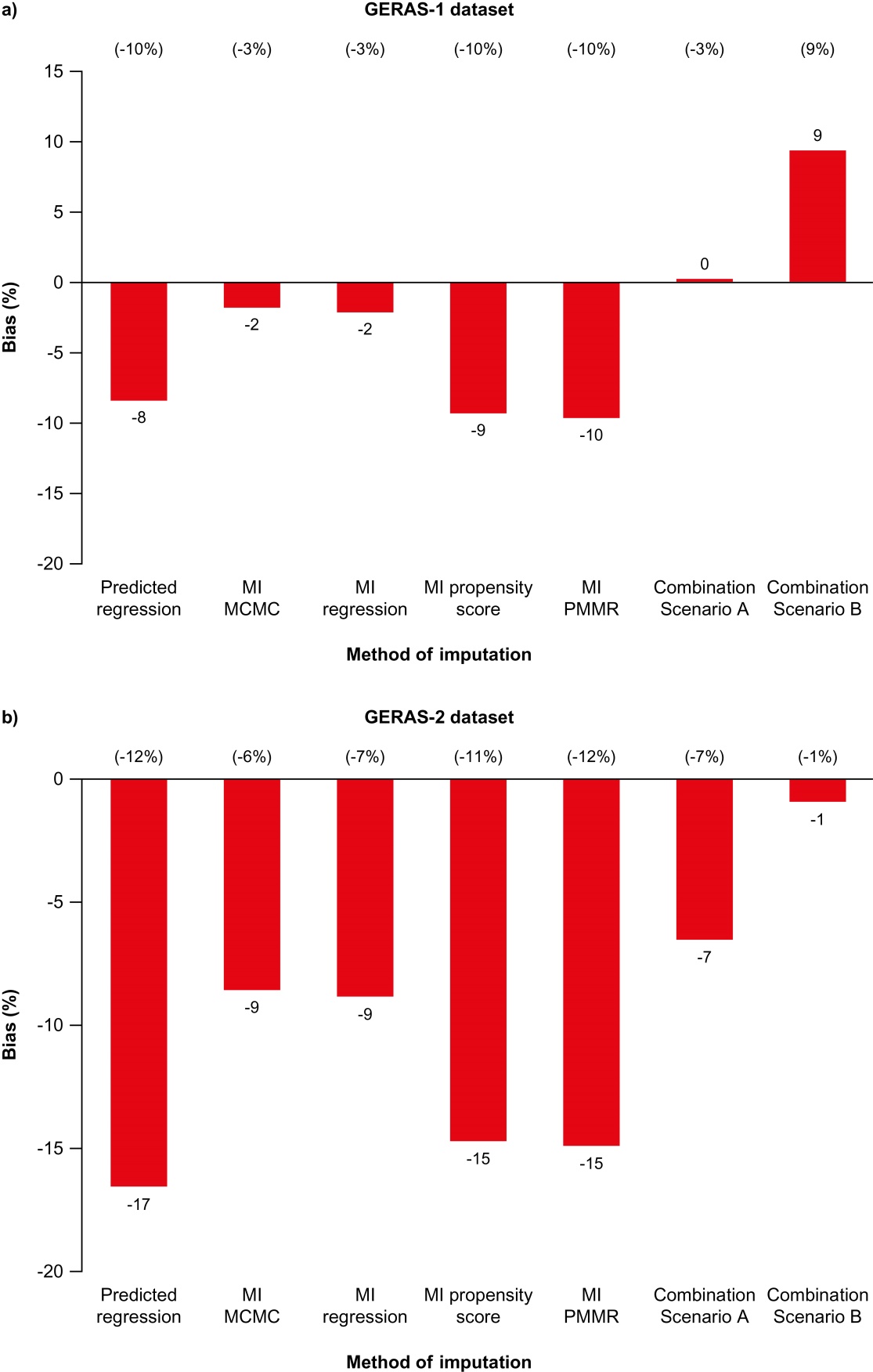


All values given above/below the columns have been rounded to the nearest whole number.

For comparison, the percentage bias in mean costs for each method in the primary simulations (without unmeasured confounding) is provided in brackets at the top of each figure.

MCMC = Markov Chain Monte Carlo; MI = multiple imputation; PMMR = predictive mean matching regression

**Supplemental Figure** **2** Impact of unmeasured confounders on percentage bias in mean costs for selected imputation methods with the MAR dataset


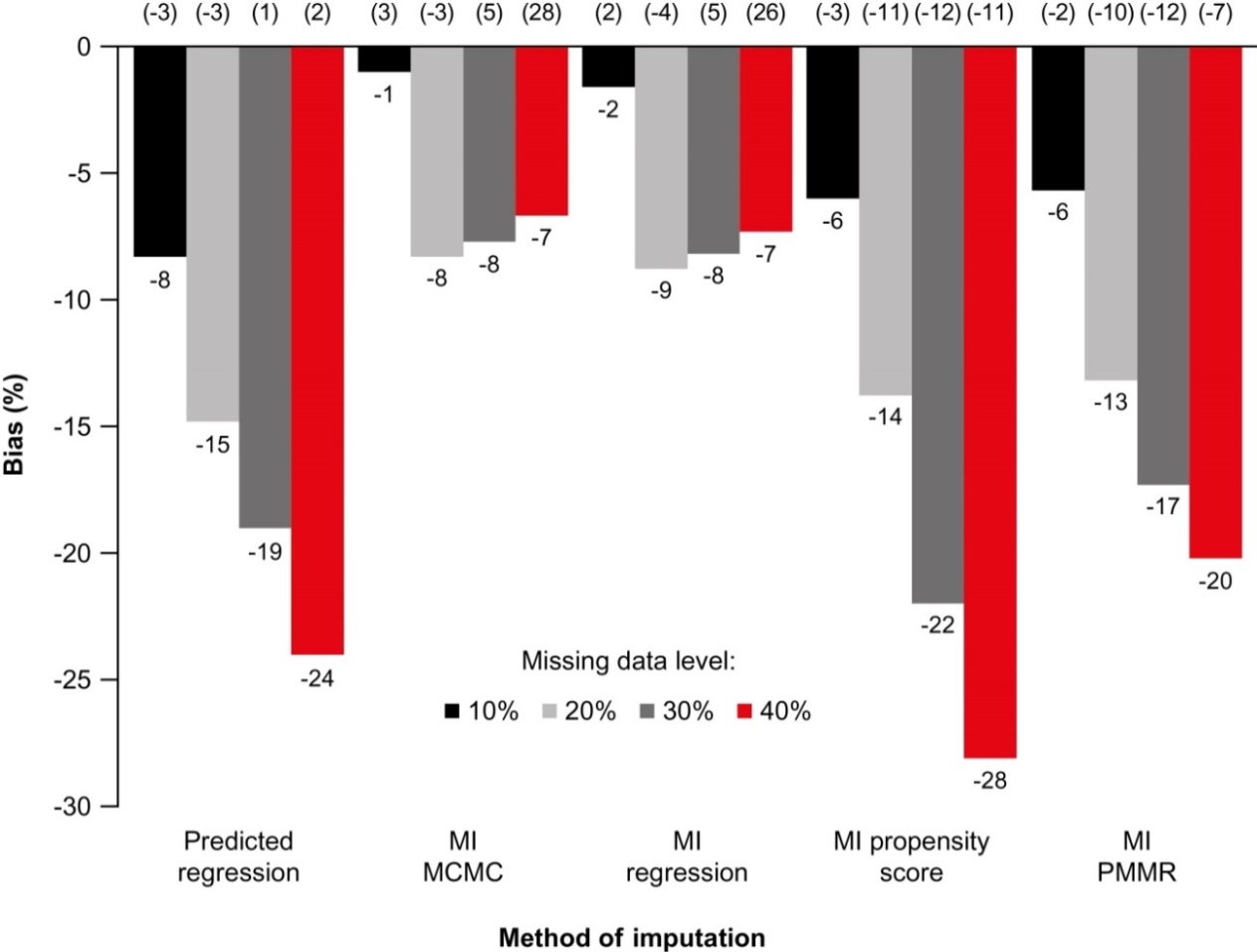


All values given above/below the columns have been rounded to the nearest whole number.

For comparison, the percentage bias in mean costs for each method in the primary simulation (without unmeasured confounding) is provided in brackets at the top of the figure

MCMC = Markov Chain Monte Carlo; MI = multiple imputation; PMMR = predictive mean matching regression

### Different sample sizes

Supplemental Figure 3 shows that when the sample sizes are smaller (n=500 and n=100), the % bias in mean costs is consistent with that seen in the primary analysis with the larger sample size (n=1497), even among the MI methods that assume costs are normally distributed (MI MCMC and MI regression). This suggests that even at sample sizes of 100, the assumptions around the distribution of costs do not affect the estimates; this is probably because the assumption on normality for the MI methods is required only for the data that are missing. However, the standard error (SE) is affected by the sample size. For example, the ‘true’ SE for the complete sample increases from 62 for the full sample (n=1497) to 106 for the n=500 sample, to 227 for the n=100 sample.

**Supplemental Figure 3** Effect of sample size on percentage bias in mean costs for selected imputation methods on datasets GERAS-1 and GERAS-2


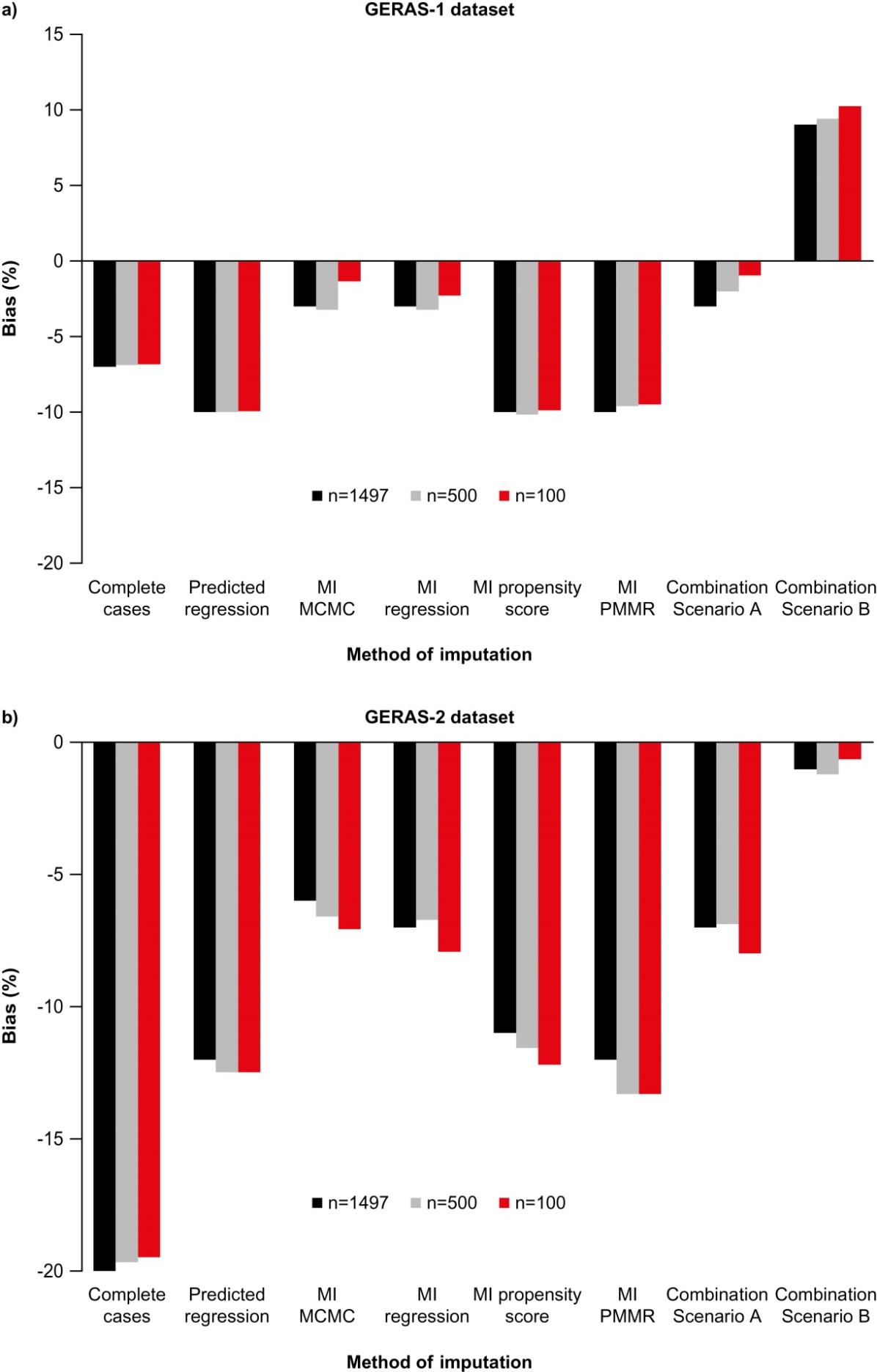


MCMC = Markov Chain Monte Carlo; MI = multiple imputation; PMMR = predictive mean matching regression

Supplemental Figure 4 shows the effect of sample size on the value of the standard error estimator relative to the sampling standard error (SEE/SSE) for each imputation method for the GERAS-1 and GERAS-2 datasets. For all sample sizes, Combination Scenario B had a higher SEE/SSE ratio than the other imputation methods (except complete cases, where there was no imputation), which showed that these other imputation methods underestimated the SE of the mean costs. Although the % bias did not appear to be affected when the sample size was n=100, there were some replications of the simulations where the MI algorithms would not run because of too few observations; these were resolved by removing the country and Mini-Mental State Examination (MMSE) severity stratification conditions and, although this did not have an effect on these simulations, it could have an impact under different circumstances where stratification of the MI model is important.

**Supplemental Figure 4** Effect of sample size on ratio of SEE to SSE for datasets GERAS-1 and GERAS-2


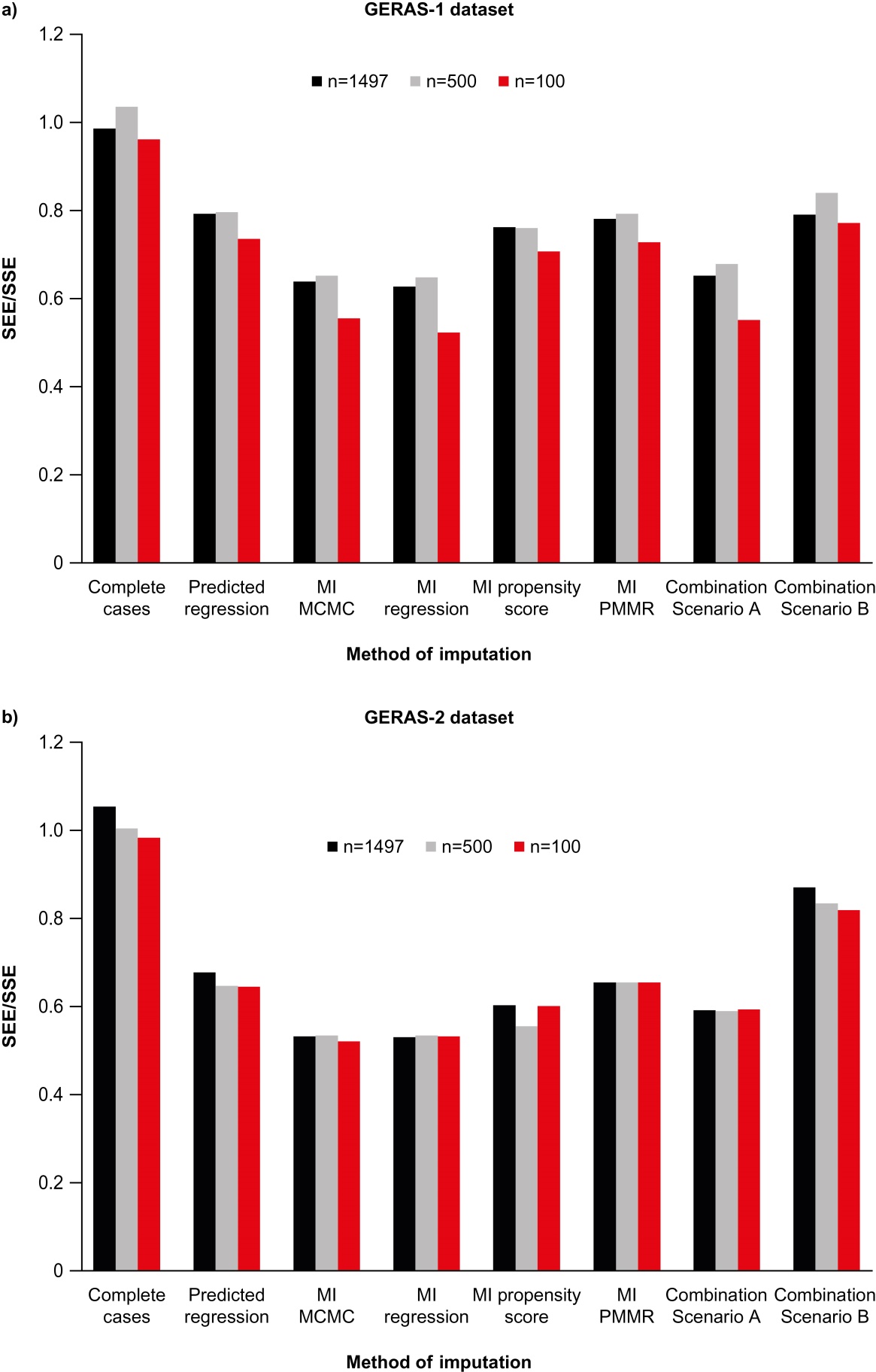


MCMC = Markov Chain Monte Carlo; MI = multiple imputation; PMMR = predictive mean matching regression; SEE = standard error estimate; SSE = sampling standard error
